# Evolutionary Analysis of Classical *HLA* Class I and II Genes Suggests That Recent Positive Selection Acted on DPB1*04∶01 in Japanese Population

**DOI:** 10.1371/journal.pone.0046806

**Published:** 2012-10-03

**Authors:** Minae Kawashima, Jun Ohashi, Nao Nishida, Katsushi Tokunaga

**Affiliations:** 1 Department of Human Genetics, Graduate School of Medicine, The University of Tokyo, Tokyo, Japan; 2 Molecular and Genetic Epidemiology, Faculty of Medicine, University of Tsukuba, Ibaraki, Japan; 3 The Research Center for Hepatitis and Immunology, National Center for Global Health and Medicine, Ichikawa, Chiba, Japan; University of Utah, United States of America

## Abstract

The human leukocyte antigen (*HLA*) genes exhibit the highest degree of polymorphism in the human genome. This high degree of variation at classical *HLA* class I and class II loci has been maintained by balancing selection for a long evolutionary time. However, little is known about recent positive selection acting on specific *HLA* alleles in a local population. To detect the signature of recent positive selection, we genotyped six *HLA* loci, *HLA-A*, *HLA-B*, *HLA-C*, *HLA-DRB1*, *HLA-DQB1*, and *HLA-DPB1* in 418 Japanese subjects, and then assessed the haplotype homozygosity (*HH*) of each *HLA* allele. There were 120 *HLA* alleles across the six loci. Among the 80 *HLA* alleles with frequencies of more than 1%, DPB1*04∶01, which had a frequency of 6.1%, showed exceptionally high *HH* (0.53). This finding raises the possibility that recent positive selection has acted on DPB1*04∶01. The DPB1*04∶01 allele, which was present in the most common 6-locus *HLA* haplotype (4.4%), A*33∶03-C*14∶03-B*44∶03-DRB1*13∶02-DQB1*06∶04-DPB1*04∶01, seems to have flowed from the Korean peninsula to the Japanese archipelago in the Yayoi period. A stochastic simulation approach indicated that the strong linkage disequilibrium between DQB1*06∶04 and DPB1*04∶01 observed in Japanese cannot be explained without positive selection favoring DPB1*04∶01. The selection coefficient of DPB1*04∶01 was estimated as 0.041 (95% credible interval 0.021–0.077). Our results suggest that DPB1*04∶01 has recently undergone strong positive selection in Japanese population.

## Introduction

The crucial immunological function of human leukocyte antigen (HLA) molecules is to present pathogen-derived antigenic peptides to T lymphocytes [1]. The HLA proteins are encoded by genes in the major histocompatibility complex region, which spans approximately 4 megabases (Mb) on the short arm of chromosome 6 (6p21.3) and includes the most polymorphic loci in the human genome [2]. A remarkable feature of the classical *HLA* class I and class II genes is the high degree of polymorphism. More than 1,750 *HLA-A*, 2,330 *HLA-B*, 1,300 *HLA-C*, 1,060 *HLA-DRB1*, 160 *HLA-DQB1*, and 150 *HLA-DPB1* alleles have been reported (IMGT/HLA database; http://www.ebi.ac.uk/imgt/hla/).

Positive selection has been shown as a driving force for the high degree of polymorphism at *HLA* loci [3], [4]. The *HLA* genes show three remarkable signatures of positive selection: (1) the rate of nonsynonymous (amino acid altering) nucleotide substitution is substantially higher than that of synonymous substitution at antigen-recognition sites [5], [6], (2) there are trans-species polymorphisms (i.e., similar alleles are present in multiple species) [7], and (3) there is a significant excess of heterozygosity [8], [9]. Balancing selection, including overdominant selection and frequency-dependent selection, can easily account for these observations [3], [4].

A number of studies have reported common long-range *HLA* haplotypes [10]–[16]. The extended length of common haplotype is a key feature of recent positive selection [17], [18]. The *HLA* alleles on long-range haplotypes may have been subject to recent positive selection. In this study, to identify the signature of recent positive selection that has acted on specific *HLA* alleles in a local (i.e., geographically restricted) population, we investigated the allele frequencies and haplotype frequencies at *HLA-A*, *HLA-C*, *HLA-B*, *HLA-DRB1*, *HLA-DQB1*, and *HLA-DPB1* in 418 Japanese individuals. Our theoretical and computer simulation analyses suggested that DPB1*04∶01 has recently undergone strong positive selection in Japanese population.

## Results

### *HLA* Class I and Class II Alleles in Japanese

The genotypes of six *HLA* genes (three class I and three class II genes) were determined for each of 418 Japanese individuals. The frequencies of the 67 alleles found at the three *HLA* class I genes are listed in Table 1. Of the 17 *HLA-A* alleles, two–A*02∶01 and A*24∶02–had frequencies higher than 10% (10.2 and 37.7 percent, respectively). Of the 17 *HLA-C* alleles, four–C*01∶02, C*03∶03, C*03∶04, and C*07∶02–had frequencies higher than 10%: 16.5, 13.5, 12.6, and 14.5 percent, respectively. There were 33 *HLA-B* alleles, and not one had an allele frequency greater than 10%. The allele with the highest frequency (9.6%) was B*52∶01; this allele was followed by B*15∶01 (8.5%), B*51∶01 (8.5%), B*4403 (8.1%), and B*35∶01 (8.0%).

**Table 1:** Frequencies of *HLA* class I alleles.

HLA-A						HLA-C						HLA-B					
Allele	Count	Freq.	H^a^	HWE^b^	EW^c^	Allele	Count	Freq.	H^a^	HWE^b^	EW^c^	Allele	Count	Freq.	H^a^	HWE^b^	EW^c^
				P-val	P-val					P-val	P-val					P-val	P-val
A*01∶01	10	0.012	0.810	0.667	0.294	C*01∶02	138	0.165	0.891	0.919	0.003	B*07∶02	57	0.068	0.937	0.286	0.002
A*02∶01	85	0.102				C*01∶03	4	0.005				B*13∶01	13	0.016			
A*02∶06	61	0.073				C*03∶02	3	0.004				B*15∶01	71	0.085			
A*02∶07	23	0.028				C*03∶03	113	0.135				B*15∶07	5	0.006			
A*02∶10	2	0.002				C*03∶04	105	0.126				B*15∶11	5	0.006			
A*03∶01	4	0.005				C*04∶01	42	0.050				B*15∶18	13	0.016			
A*03∶02	1	0.001				C*05∶01	5	0.006				B*15∶27	1	0.001			
A*11∶01	80	0.096				C*06∶02	7	0.008				B*15∶28	1	0.001			
A*24∶02	315	0.377				C*07∶02	121	0.145				B*27∶04	2	0.002			
A*24∶08	1	0.001				C*07∶04	7	0.008				B*35∶01	67	0.080			
A*24∶20	10	0.012				C*08∶01	47	0.056				B*37∶01	7	0.008			
A*26∶01	67	0.080				C*08∶03	12	0.014				B*39∶01	34	0.041			
A*26∶02	12	0.014				C*12∶02	81	0.097				B*39∶04	5	0.006			
A*26∶03	22	0.026				C*12∶03	1	0.001				B*40∶01	46	0.055			
A*26∶05	1	0.001				C*14∶02	50	0.060				B*40∶02	57	0.068			
A*31∶01	66	0.079				C*14∶03	69	0.083				B*40∶03	7	0.008			
A*33∶03	76	0.091				C*15∶02	31	0.037				B*40∶06	34	0.041			
												B*40∶52	1	0.001			
												B*44∶02	5	0.006			
												B*44∶03	68	0.081			
												B*46∶01	38	0.045			
												B*48∶01	22	0.026			
												B*51∶01	71	0.085			
												B*51∶02	4	0.005			
												B*52∶01	80	0.096			
												B*54∶01	64	0.077			
												B*55∶02	20	0.024			
												B*55∶04	1	0.001			
												B*56∶01	5	0.006			
												B*56∶03	2	0.002			
												B*58∶01	3	0.004			
												B*59∶01	16	0.019			
												B*67∶01	11	0.013			

a Heterozygosity.

b Hardy-Weinberg equilibrium test.

c Ewens-Watterson test.

The frequencies of 53 alleles at three *HLA* class II genes are listed in Table 2. Of the 27 alleles at the *HLA-DRB1* locus, two–DRB1*09∶01 and DRB1*04∶05–had frequencies of more than 10% (15.2% and 14.6%, respectively), and five–DRB1*15∶02 (8.4%), DRB1*15∶01 (8.0%), DRB1*13∶02 (7.8%), DRB1*08∶03 (7.5%), and DRB1*01∶01 (6.8%)–were also common. Of the 14 alleles at *HLA-DQB1*, four–DQB1*03∶03, DQB1*06∶01, DQB1*04∶01, and DQB1* 03∶01–were observed at frequencies of greater than 10% (15.9%, 15.9%, 14.6%, and 11.8%, respectively). There were four other common alleles at *HLA-DQB1*–DQB1*03∶02 (9.2%), DQB1*06∶02 (7.8%), DQB1*05∶01, and DQB1*06∶04 (7.5%). Of the six *HLA* loci genotyped, *HLA-DPB1* had the fewest alleles with just 12. The DPB1*05∶01 (38.5%) and DPB1*02∶01 (25.1%) alleles were the most frequent alleles at this locus.

**Table 2:** Frequencies of *HLA* class II alleles.

*HLA-DRB1*						*HLA-DQB1*						*HLA-DPB1*					
Allele	Count	Freq.	H^a^	HWE^b^	EW^c^	Allele	Count	Freq.	H^a^	HWE^b^	EW^c^	Allele	Count	Freq.	H^a^	HWE^b^	EW^c^
				P-val	P-val					P-val	P-val					P-val	P-val
DRB1*01∶01	57	0.068	0.918	0.247	0.013	DQB1*02∶01	1	0.001	0.885	0.222	0.001	DPB1*02∶01	210	0.251	0.765	0.398	0.225
DRB1*03∶01	1	0.001				DQB1*03∶01	99	0.118				DPB1*02∶02	35	0.042			
DRB1*04∶01	10	0.012				DQB1*03∶02	77	0.092				DPB1*03∶01	36	0.043			
DRB1*04∶03	24	0.029				DQB1*03∶03	133	0.159				DPB1*04∶01	51	0.061			
DRB1*04∶04	2	0.002				DQB1*04∶01	122	0.146				DPB1*04∶02	83	0.099			
DRB1*04∶05	122	0.146				DQB1*04∶02	26	0.031				DPB1*05∶01	322	0.385			
DRB1*04∶06	28	0.033				DQB1*05∶01	63	0.075				DPB1*06∶01	5	0.006			
DRB1*04∶07	1	0.001				DQB1*05∶02	17	0.020				DPB1*09∶01	65	0.078			
DRB1*04∶10	12	0.014				DQB1*05∶03	30	0.036				DPB1*13∶01	12	0.014			
DRB1*08∶02	32	0.038				DQB1*06∶01	133	0.159				DPB1*14∶01	10	0.012			
DRB1*08∶03	63	0.075				DQB1*06∶02	65	0.078				DPB1*19∶01	5	0.006			
DRB1*09∶01	127	0.152				DQB1*06∶03	5	0.006				DPB1*41∶01	2	0.002			
DRB1*10∶01	6	0.007				DQB1*06∶04	63	0.075									
DRB1*11∶01	23	0.028				DQB1*06∶09	2	0.002									
DRB1*12∶01	30	0.036															
DRB1*12∶02	18	0.022															
DRB1*13∶01	5	0.006															
DRB1*13∶02	65	0.078															
DRB1*14∶02	1	0.001															
DRB1*14∶03	11	0.013															
DRB1*14∶05	17	0.020															
DRB1*14∶06	13	0.016															
DRB1*14∶07	3	0.004															
DRB1*14∶54	26	0.031															
DRB1*15∶01	67	0.080															
DRB1*15∶02	70	0.084															
DRB1*16∶02	2	0.002															

a Heterozygosity.

b Hardy-Weinberg equilibrium test.

c Ewens-Watterson test.

Of the six *HLA* loci examined, the *HLA-B* locus showed the highest heterozygosity (0.937), and *HLA-DPB1* showed the lowest (0.765) (Tables 1 and 2). None of the *HLA* class I or II loci exhibited significant deviation from HWE. Results of a Ewens-Watterson neutrality test [19], [20] of *HLA* allele frequencies in this study population revealed that the observed distributions of allele frequencies at *HLA-C* (*P* = 0.003), *HLA-B* (*P* = 0.002), *HLA-DRB1* (*P* = 0.013), and *HLA-DQB1* (*P* = 0.001) differed significantly (i.e., there was excess heterozygosity) from the distributions expected based on the assumption of neutrality, whereas there was no significant difference between the expected and observed distributions of allele frequencies at *HLA-A* or *HLA-DPB1* (Tables 1 and 2).

### Pairwise LD between *HLA* Alleles

**Figure 1:**
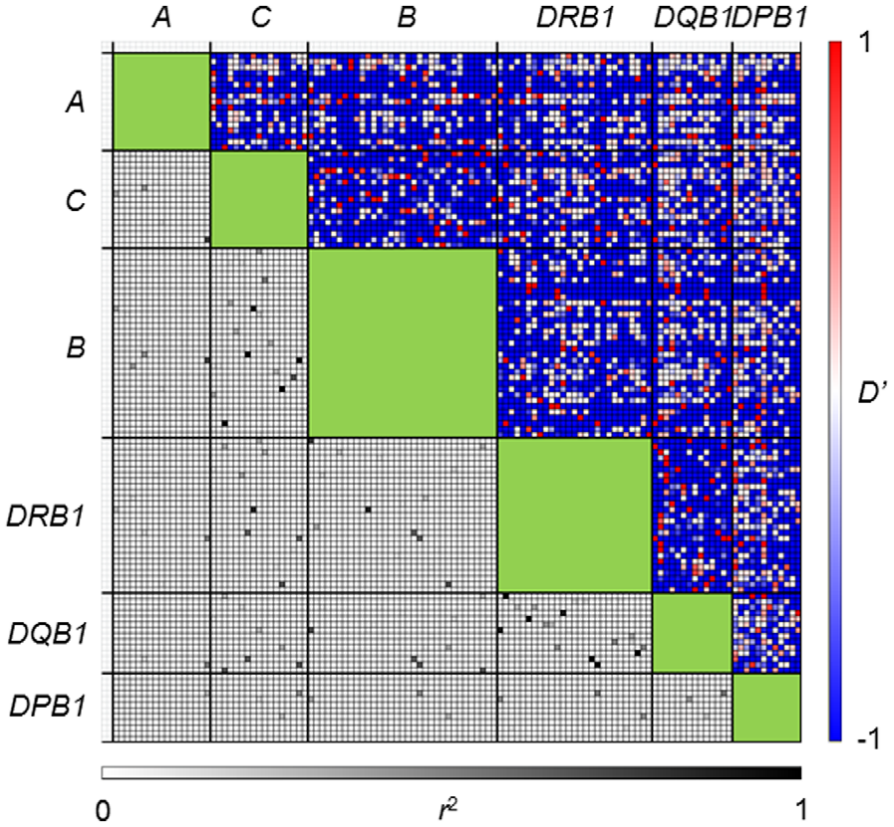
Pairwise estimates of LD parameters, |*D*’| (upper diagonal) and *r* ^2^ (lower diagonal) for every pair of *HLA* alleles. The name of each allele is presented in Data S1.

**Figure 2:**
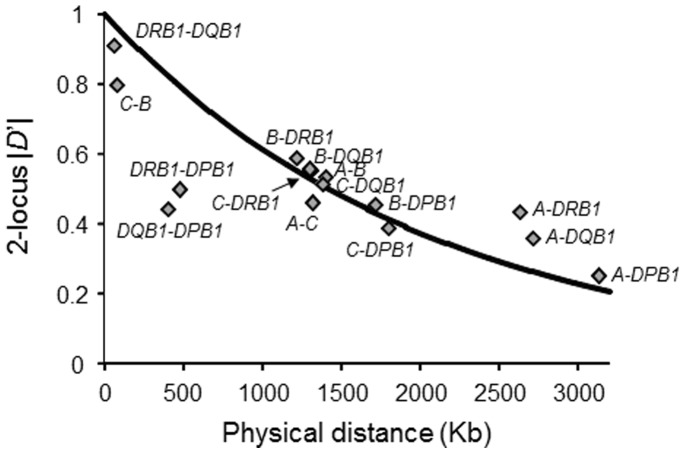
Relationship between two-locus |*D*’| and physical distance (Kb). A solid-line curve, 
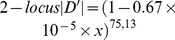
, was obtained using the least-squares method, where *x* represents the physical distance (Kb). The recombination rate in the *HLA* region was assumed to be 0.67 cM/Mb [27]. Spearman’s rank correlation coefficient between 2-locus |*D*’| and the physical distance was −0.8607 (*P*<0.0001).

The pairwise linkage disequilibrium (LD) parameters, *r* ^2^ and |*D*’| [21], for each possible pair of two *HLA* alleles were estimated (Figure 1 and Data S1). Most alleles at *HLA-A* were not in strong LD with any of the alleles at the other loci because the physical distance from *HLA-A* to each of the other loci is large. To evaluate the relative strength of LD between two *HLA* loci, 2-locus *r* ^2^ and 2-locus |*D*’| (see Materials and Methods for details), were calculated based on the pairwise LD parameters for all the allelic pairs (Table S1). The values of 2-locus |*D*’| for *HLA-C* and *HLA-B* (|*D*’| = 0.91) and for *HLA-DRB1* and *HLA-DQB1* (|*D*’| = 0.80) were high, whereas the lowest 2-locus |*D*’| value was observed for *HLA-A* and *HLA-DPB1* (|*D*’| = 0.25). These values reflected the physical distances between the respective loci. The values of 2-locus |*D*’| for *HLA-DRB1* and *HLA-DPB1* and for *HLA-DQB1* and *HLA-DPB1* were relatively low compared to the values for the other pairs (Figure 2). These low values probably result from the recombination hotspot in the *HLA* class II region [22]–[24].

### Major 6-locus *HLA* Haplotypes in Japanese

Frequencies of multi-locus haplotypes were estimated using the PHASE program [25], [26] (Table 3 and Tables S2, S3, S4, S5). In 418 Japanese subjects (i.e., 839 chromosomes), 489 different 6-locus *HLA* haplotypes were inferred. Based on the frequencies of 6-locus *HLA* haplotypes, the probability of selecting two identical 6-locus *HLA* haplotypes at random from the Japanese population was estimated as 0.0075. Six 6-locus *HLA* haplotypes had frequencies higher than 1% (Table 3). Of these, A*33∶03-C*14∶03-B*44∶03-DRB1*13∶02-DQB1*06∶04-DPB1*04∶01 was the most common (4.4%).

**Table 3:** Estimated frequencies of 6-locus *HLA* haplotypes.

Association						# of haplotypes^a^	HF^b^
A*33∶03	C*14∶03	B*44∶03	DRB1*13∶02	DQB1*06∶04	DPB1*04∶01	37	0.044
A*24∶02	C*12∶02	B*52∶01	DRB1*15∶02	DQB1*06∶01	DPB1*09∶01	33	0.039
A*24∶02	C*07∶02	B*07∶02	DRB1*01∶01	DQB1*05∶01	DPB1*04∶02	29	0.035
A*24∶02	C*01∶02	B*54∶01	DRB1*04∶05	DQB1*04∶01	DPB1*05∶01	13	0.016
A*24∶02	C*12∶02	B*52∶01	DRB1*15∶02	DQB1*06∶01	DPB1*02∶01	12	0.014
A*11∶01	C*04∶01	B*15∶01	DRB1*04∶06	DQB1*03∶02	DPB1*02∶01	11	0.013

a Estimated by the PHASE program version 2.1.

b Haplotype frequency.

The intensity of recombination in the *HLA* region has been estimated at 0.67 cM/Mb [27], which corresponds to a recombination fraction of approximately 2% between *HLA-A* and *HLA-DPB1*. Thus, association between the six *HLA* alleles in any 6-locus *HLA* haplotype is not generally strong due to the frequent recombination in the *HLA* region. The expected frequency of the A*33∶03-C*14∶03-B*44∶03-DRB1*13∶02-DQB1*06∶04-DPB1*04∶01 haplotype is 2.5×10^−7^ under the assumption of linkage equilibrium, which is much smaller than the observed frequency of 0.044. The strong LD among *HLA* alleles on the A*33∶03-C*14∶03-B*44∶03-DRB1*13∶02-DQB1*06∶04-DPB1*04∶01 haplotype may result from recent positive selection acting on one of *HLA* alleles on the haplotype, although other mechanisms such as neutral random genetic drift, recent admixture, recent migration, recent bottlenecks, and suppression of recombination can also cause the strong LD [10], [12], [13], [15], [16].

### Haplotype Omozygosity

Strong positive selection leads to a rapid increase in the frequency of a selected (target) allele in a population. The number of recombination events between the target allele and the surrounding polymorphic sites is limited while the advantageous allele increases in frequency; therefore, the diversity of haplotypes carrying the advantageous allele becomes low. Accordingly, strong LD is expected in the genomic region bearing the selected allele. In this study, the degree of LD for each HLA allele was measured by haplotype homozygosity (*HH*); this term is defined as the probability that any two randomly chosen samples of haplotype bearing a focal *HLA* allele have the same 6-locus *HLA* haplotype. Like *EHH* [17], a high *HH* value can be regarded as a signature of recent positive selection acting on a focal *HLA* allele.

**Figure 3:**
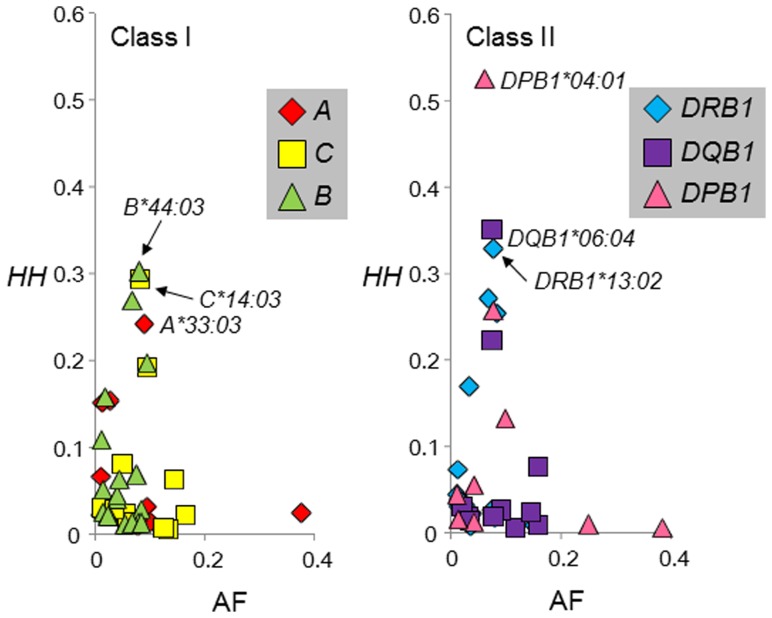
Haplotype homozygosity (*HH*) × allele frequency (AF) of each *HLA* allele. The left and right panels show *HH* values of *HLA* class I alleles and *HLA* class II alleles, respectively. The class I alleles were designated as follows: *HLA-A* (red diamond), *HLA-C* (yellow square), and *HLA-B* (green triangle); the class II alleles were designated as follows: *HLA*-*DRB1* (blue diamond), *HLA-DQB1* (purple square), and *HLA-DPB1* (pink triangle). In both panels, only *HH* values of alleles with frequencies of more than 0.01 are shown.

To detect *HLA* alleles that have been subject to recent positive selection, *HH* was calculated for each allele based on the estimated number of 6-locus haplotypes in 418 Japanese subjects. Of the 80 *HLA* alleles that had frequencies of more than 1%, one allele at each class I locus (A*33∶03, C*14∶03, and B*44∶03) had the highest *HH* for that locus; similarly, one allele at each class II locus (DRB1*13∶02, DQB1*06∶04, and DPB1*04∶01) had the highest *HH* for that locus (Figure 3). These six *HLA* alleles made up the 6-locus haplotype, A*33∶03-C*14∶03-B*44∶03-DRB1*13∶02-DQB1*06∶04-DPB1*04∶01, with the highest frequency in this Japanese population (Table 3).

The *HH* values are generally reduced by loci with high heterozygosity. Therefore, it was relatively difficult for an allele at *HLA*-*DPB1* to show high *HH*, because heterozygosities at the other loci are high. Nevertheless, the DPB1*04∶01 allele, which had a population frequency of 6.1%, showed the highest *HH* value (0.53) of the 80 *HLA* alleles with frequencies higher than 1% (Figure 3). The values of *HH* of the remaining 79 *HLA* alleles were less than 0.33. This finding suggests that DPB1*04∶01 had undergone recent positive selection in Japan. The large *HH* values of the five other alleles (A*33∶03, C*14∶03, B*44∶03, DRB1*13∶02, and DQB1*06∶04) in this 6-locus *HLA* haplotype (i.e., A*33∶03-C*14∶03-B*44∶03-DRB1*13∶02-DQB1*06∶04-DPB1*04∶01) appear to be due to the hitchhiking effect of DPB1*04∶01.

**Figure 4:**
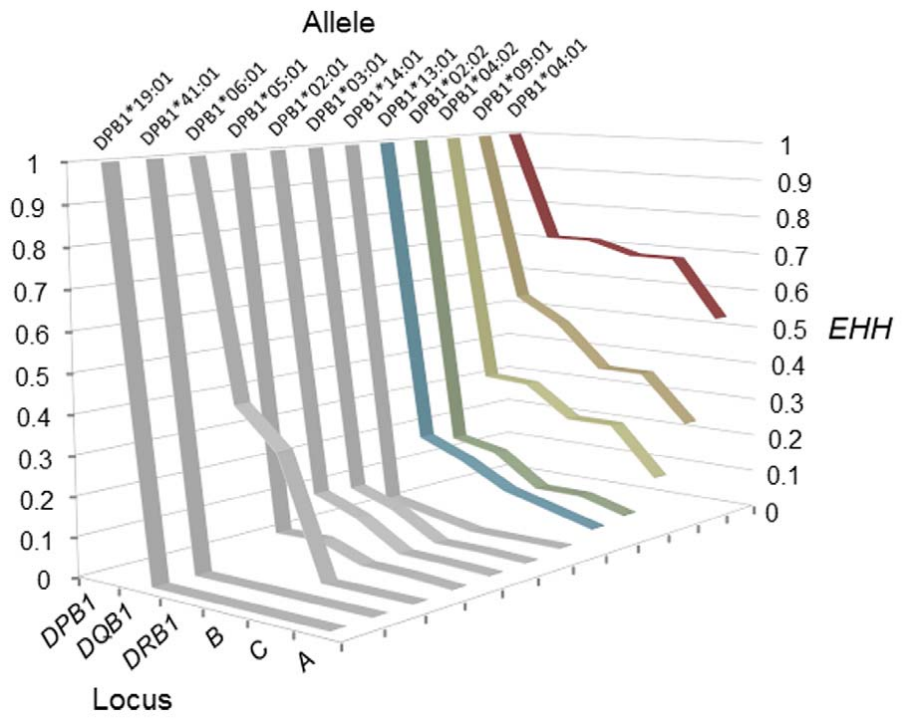
Extended *HH* (*EHH*) × relative locus position for12 *HLA-DPB1* alleles.

To investigate the effect of recombination on the decay of the A*33∶03-C*14∶03-B*44∶03-DRB1*13∶02-DQB1*06∶04-DPB1*04∶01 haplotype, the value of extended haplotype homozygosity (*EHH*) was calculated for DPB1*04∶01 (Figure 4). Although the *EHH* of DPB1*04∶01 was reduced at *HLA-DQB1*, the decrease in *EHH* was almost negligible at *HLA-DRB1*, *HLA-B*, and *HLA-C* loci; these findings indicate that, in this haplotype, recombination mainly has occurred between DQB1*06∶04 and DPB1*04∶01.

### Origin of DPB1*04∶01 in Japanese

DPB1*04∶01 is common (>30%) in European populations [9], [28], whereas the frequency of DPB1*04∶01 is 6.1% in Japanese (Table 2). Given the worldwide distribution of DPB1*04∶01, it is unlikely that DPB1*04∶01 originated in Japan. DPB1*04∶01 seems to have entered Japan. Archaeological studies of Japanese history have suggested that the Yayoi people came from the Korean peninsula circa 300 B.C., and mixed with the indigenous Jomon people. A recent large-scale survey of single nucleotide polymorphisms (SNPs) on autosomal chromosomes [29] revealed that most people presently inhabiting mainland Japan are genetically closer to Koreans than to Ryukuans. Ryukuans are considered to be more pure descendants of the Jomon people than are mainland Japanese. These observations indicate that a large population of Yayoi people migrated from the Korean peninsula. Although the frequency of the A*33∶03-C*14∶03-B*44∶03-DRB1*13∶02-DQB1*06∶04-DPB1*04∶01 haplotype in Koreans has not been reported, DPB1*04∶01, which was carried by A*33∶03-C*14∶03-B*44∶03-DRB1*13∶02-DQB1*06∶04-DPB1*04∶01, appears to have derived from the Korean population because the A*33∶03-C*14∶03-B*44∶03-DRB1*13∶02-DQB1*06∶04 and DRB1*13∶02-DQA1*01∶02-DQB1*06∶04-DPB1*04∶01 haplotypes are observed at the frequencies of 4.2% and 4.7% in Korean populations [28], [30], [31]. These and similar haplotypes have not been reported in other Asian populations (http://www.allelefrequencies.net) [28].

**Figure 5:**
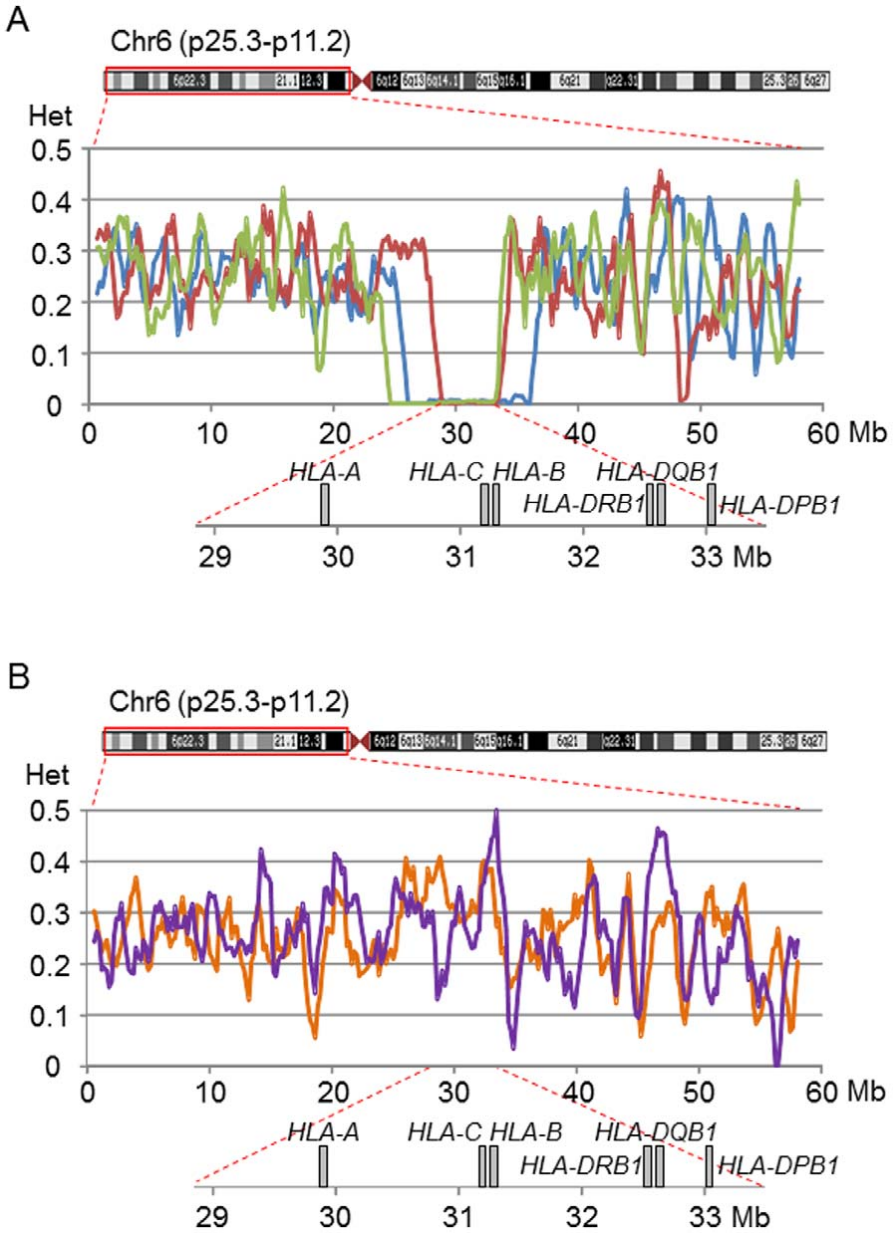
Individual heterozygosity of each subject with the most common 6-locus *HLA* haplotype. The individual heterozygosity in the genomic region on the short arm of chromosome 6 was assessed using the sliding window analysis; in this analysis, the window and step sizes were set to be 1 Mb and 200 kb, respectively. The individual heterozygosity was defined as a proportion of heterozygous SNPs to SNPs genotyped in a single subject. This analysis was performed for five Japanese subjects with the A*33∶03-C*14∶03-B*44∶03-DRB1*13∶02-DQB1*06∶04-DPB1*04∶01 haplotype: (A) three of these five subjects were homozygous for this haplotype (blue, red, and green) and (B) two subjects had the heterozygous genotypes of the A*33∶03-C*14∶03-B*44∶03-DRB1*13∶02-DQB1*06∶04-DPB1*04∶01 haplotype and the A*24∶02-C*07∶02-B*07∶02-DRB1*01∶01–DQB1*05∶01-DPB1*04∶02 haplotype (orange) and of the A*33∶03-C*14∶03-B*44∶03-DRB1*13∶02-DQB1*06∶04-DPB1*04∶01 haplotype and the A*24∶02-C*12∶02-B*52∶01-DRB1*15∶02-DQB1*06∶01-DPB1*09∶01 haplotype (purple).

If the A*33∶03-C*14∶03-B*44∶03-DRB1*13∶02-DQB1*06∶04-DPB1*04∶01 haplotype has a single origin, the current genetic diversity of this haplotype must be low. To assess the genetic diversity of this haplotype, we performed a sliding window analysis of individual heterozygosity, defined as a proportion of heterozygous SNPs to all SNPs in the window (Figure 5). Reduced individual heterozygosity was only found in the *HLA* region on the short arm of chromosome 6 in all the three subjects that were homozygous for the A*33∶03-C*14∶03-B*44∶03-DRB1*13∶02-DQB1*06∶04-DPB1*04∶01 haplotype (Figure 5A); in contrast, such a reduction was not observed in two subjects that were heterozygous for this haplotype (Figure 5B). Furthermore, three subjects that were homozygous for the A*33∶03-C*14∶03-B*44∶03-DRB1*13∶02-DQB1*06∶04-DPB1*04∶01 haplotype shared the same SNP haplotype that spanned more than 4 Mb in the *HLA* region (Figure 5A). These observations suggest that the A*33∶03-C*14∶03-B*44∶03-DRB1*13∶02-DQB1*06∶04-DPB1*04∶01 haplotype in Japanese has a single origin, and has not been generated repeatedly by recombination.

### Computer Simulation

**Figure 6:**
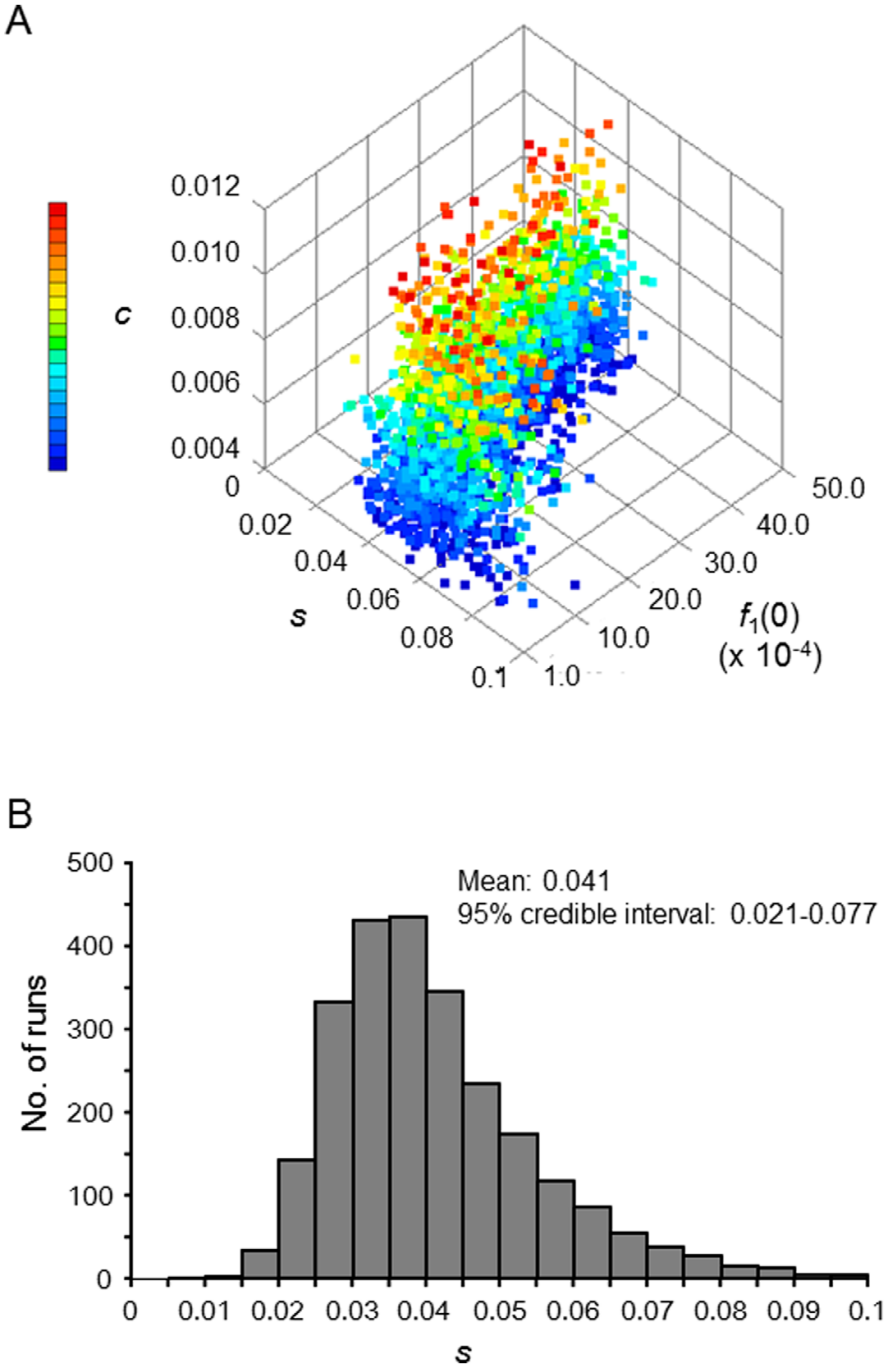
Estimation of model parameters for positive selection acting on DPB1*04∶01. The recombination rate (*c*), initial haplotype frequency (*f*_1_(0)), and selection coefficient (*s*), were estimated by comparing the four haplotype frequencies observed in our study population with the respective values predicted via simulation. (A) Posterior distributions of the three parameters that produced simulated data that resemble the observed data. (B) Frequency distribution of *s* accepted in simulation runs. The mean and 95% credible interval of *s* are 0.041 and 0.021−0.077.

The analysis of *EHH* revealed that the reduction in *EHH* for DPB1*04∶01 resulted from recombination between DQB1*06∶04 and DPB1*04∶01 that inhabited the A*33∶03-C*14∶03-B*44∶03-DRB1*13∶02-DQB1*06∶04-DPB1*04∶01 haplotype (Figure 4). Therefore, the relationship between DQB1*06∶04 and DPB1*04∶01 was focused in the following analyses. The high *HH* and *EHH* values of DPB1*04∶01 (Figures 3 and 4) may merely reflect that a neutral random genetic drift, rather than a recent positive selection, occurred after the Yayoi people reached the Japanese archipelago (300 B.C. or 2300 years ago). To assess this possibility, we conducted a computer simulation assuming a two-locus two-allele model in which changes in the frequency of four haplotypes carrying DPB1*04∶01 or non-DPB1*04∶01 alleles at the *HLA-DPB1* locus and DQB1*06∶04 or non-*DQB1∶06:04* alleles at the *HLA-DQB1* locus were evaluated. In the simulation, the values of three parameters: selection intensity, *s*, recombination rate, *c*, and frequency of DQB1*06∶04-DPB1*04∶01 haplotype, *f*_1_(0), in the beginning of the Yayoi period were drawn by a random number generator in every run. Haplotype frequencies were subject to change based on a stochastic model of positive selection, recombination, and random genetic drift. Dominant selection was assumed for DPB1*04∶01, and, for the sake of simplicity, no selection (i.e., selectively neutral) was assumed for all alleles at the *DQB1* locus. The rejection method [18], [32], [33] was applied to accept only simulation runs that gave results similar to the observed values (see Materials and Methods for details). The uniform distribution was used for each parameter as a prior distribution (see Materials and Methods for detail). Figure 6A shows 2,500 parameter sets (i.e., posterior distributions) that were accepted in these simulations. The posterior distribution of the initial frequency of DQB1*06∶04-DPB1*04∶01 haplotype was similar to the prior one, whereas the posterior distributions of selection intensity and recombination rate were different from the prior ones. In the posterior distribution, *s* ranged from 0.009 to 0.098, and the mean and 95% credible interval of *s* were 0.041 and 0.021−0.077, respectively (Figure 6B). It should be noted that neutral random genetic drift (i.e., *s*≈0) did not yield the results similar to the observed values. The findings from the simulations indicated that DPB1*04∶01 has been subject to relatively strong positive selection in Japanese since the Yayoi period.

## Discussion

A number of *HLA* alleles have been shown to be associated with variations in immune responses to infectious diseases (e.g., human immunodeficiency virus [HIV]/AIDS, malaria, tuberculosis, hepatitis, leprosy, leishmaniasis, and schistosomiasis) caused by pathogenic microorganisms (see review by Blackwell et al. [34]). The most plausible explanation for positive selection favoring DPB1*04∶01 would be its function in resistance to infections. A recent genome-wide association study showed that the DPA1*01∶03-DPB1*04∶01 haplotype confers protection against hepatitis B virus (HBV) infection (OR = 0.57, 95% CI = 0.33–0.96) [35]. Hepatitis B is a deadly infectious disease. Acute hepatitis B, which can cause fatal complications such as fulminant hepatitis, occurs in a percentage of the people infected with HBV. Although the estimated selection coefficient of *s* (0.0254–0.0550) for DPB1*04∶01 does not seem to result solely from protection against infection with HBV, HBV infection may have been one of the key driving forces for the rapid increase in frequency of DPB1*04∶01 in the Japanese population.

Here, the analysis of *HH* was used to detect a signature of recent positive selection. The advantage of using *HH* in the analysis of *HLA* genes is that alleles with similar frequencies not only at the same *HLA* locus, but also at different loci, can be compared. This feature of analyses based on *HH* allows us to compare *HLA* alleles even within the same long-range haplotype. Since the same polymorphic markers are used for all *HLA* alleles in the calculation of *HH*, the effect of recombination on the value of *HH* can be well controlled. However, the *HH* analysis has a disadvantage in that the empirical distribution of *HH* value has to be obtained from only those alleles that are in the targeted region. Therefore, unlike conventional long-range haplotype tests based on *EHH* values [17], [36], the statistical test based on *HH* values cannot be performed using genome-wide data. Nevertheless, *HH*-based test is thought to be suitable for analysis of *HLA* genes because each locus has a number of alleles to be examined and strong LD exists between alleles even at distant loci. The use of *HH* in the analysis of various human populations would help us to detect other *HLA* alleles that have been subject to geographically-restricted positive selection and to understand the role of *HLA* genes in the adaptation of human population to local environments over evolutionary time.

To estimate the selection coefficient of DPB1*04∶01, we used a simple two-locus two-allele genetic model that was based on two assumptions, directional selection at *DPB1* and selective neutrality at *HLA-DQB1*. The problem associated with the use of this model was that the Ewens-Watterson test revealed that the allele frequency distribution at *HLA-DQB1* in this study population deviated significantly from that expected under neutrality (Table 2); therefore, the assumption of selective neutrality at *HLA-DQB1* may not be valid. If balancing selection is operating at *HLA-DQB1*, the allele frequency of DQB1*06∶04 is maintained at a certain frequency, and the change in the allele frequency of DPB1*04∶01 must be influenced by this selection at *HLA-DQB1*, although the effect of balancing selection at *HLA-DQB1* on the estimation of *s* is considered to be much smaller than that of directional selection favoring DPB1*04∶01.

In this study, six *HLA* loci were investigated in 418 Japanese subjects. Of *HLA* alleles with high population frequencies, DPB1*04∶01, which was present in the most common 6-locus *HLA* haplotype spanning more than 4 Mb, showed exceptionally high *HH*. A computer simulation estimated the selection coefficient of DPB1*04∶01 as 0.041. Taken together with high *HH* value of DPB1*04∶01, we conclude that DPB1*04∶01 has recently undergone strong positive selection in Japanese population.

## Materials and Methods

### Subjects

All 418 individuals investigated in this study were unrelated Japanese adults living in Tokyo or neighboring areas. The genomic DNAs were extracted from peripheral blood samples using a commercial kit (QIAamp Blood Kit [Qiagen, Hilden, Germany]). All blood and DNA samples were de-identified. Verbal informed consent was obtained from all the participants before 1990. In this study, written informed consent was not obtained because the blood sampling was conducted before the “Ethical Guidelines for Human Genome and Genetic Sequencing Research” were established in Japan. Under the condition that DNA sample is permanently de-linked from the individual, this study was approved by the Research Ethics Committee of the Faculty of Medicine, University of Tokyo.

### HLA Typing

DNA typing of *HLA* alleles was performed by HLA LABORATORY (Kyoto, Japan) using a Luminex Multi-Analyte profiling system (xMAP; Luminex, Austin, TX, USA) [37].

### SNP Typing

Five Japanese subjects who had at least one A*33∶03-C*14∶03-B*44∶03-DRB1*13∶02-DQB1*06∶04-DPB1*04∶01 haplotype were genotyped using the Axiom™ Genome-Wide ASI 1 Array Plate (Affymetrix Inc., Santa Clara, CA, USA). Of five subjects, three subjects were homozygous for the A*33∶03-C*14∶03-B*44∶03-DRB1*13∶02-DQB1*06∶04-DPB1*04∶01 haplotype and two subjects had the heterozygous genotypes of the A*33∶03-C*14∶03-B*44∶03-DRB1*13∶02-DQB1*06∶04-DPB1*04∶01 haplotype and the A*24∶02-C*07∶02-B*07∶02-DRB1*01∶01-DQB1*05∶01-DPB1*04∶02 haplotype and of the A*33∶03-C*14∶03-B*44∶03-DRB1*13∶02-DQB1*06∶04-DPB1*04∶01 haplotype and the A*24∶02-C*12∶02-B*52∶01-DRB1*15∶02-DQB1*06∶01-DPB1*09∶01 haplotype.

### Statistical Analysis

Deviation from HWE for each *HLA* locus was tested using an exact test available in a web-based software, Genepop 4.0.10 [38]. Using Arlequin version 3.5 [39], the Ewens-Watterson test [40], which is based on Ewens sampling theory of neutral alleles [19], was performed to assess whether the observed distribution of allele frequencies at each *HLA* locus was different from an expectation that was based on neutrality.

To evaluate the degree of LD between *HLA* alleles, values of *r* ^2^ and *D*’ [21] for all pairwise combinations of *HLA* alleles were calculated based on the haplotype frequencies estimated using the expectation maximization algorithm [20]. Here, each *HLA* allele was regarded as a single nucleotide polymorphism (SNP). For example, the A*01∶01 allele and the other alleles at the *HLA-A* locus were designated as “A” and “G”, respectively. Accordingly, the algorithm for the estimation of haplotype frequencies for two loci, each with two alleles, could be applied to the *HLA* loci with multiple alleles for the purposes of these pairwise comparisons.

The LD parameter, 2-locus |*D*’|, between any two *HLA* loci (locus 1 and locus 2) was calculated based on the pairwise LD parameter, *D*’*_ij_*, between *i*th allele at locus 1 and *j*th allele at locus 2 as follows: 2-locus 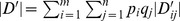, where *p_i_* and *q_j_* represent the frequencies of *i*th allele at locus 1 with *m* different alleles and *j*th allele at locus 2 with *n* different alleles. Spearman’s rank correlation coefficient between 2-locus |*D*’| and the physical distance was calculated. Assuming a model: 2-locus |*D*’| = 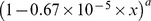, the curve fitting model parameter, *a*, was estimated using the least squares method; this method minimizes the sum-of-squared residual between an observed value and a fitted value that was determine by a model. In the above equation, the physical distance (Kb) between two loci is denoted by *x* and the recombination intensity in the *HLA* region was set at 0.65 cM/Mb [27], [41].

The phased haplotypes consisting of two or more *HLA* loci were estimated using the PHASE program version 2.1 [25], [26]. The estimated 6-locus haplotypes were further used for the calculation of extended haplotype homozygosity (*EHH*) [17] and of haplotype homozygosity (*HH*). In this study, *HH* of each *HLA* allele was defined as the probability that any two randomly chosen samples of haplotype bearing the *HLA* allele have the same 6-locus *HLA* haplotype.

A sliding window analysis of individual heterozygosity, which was defined as the proportion of heterozygous SNPs to SNPs genotyped in a single subject, was conducted to examine whether the A*33∶03-C*14∶03-B*44∶03-DRB1*13∶02-DQB1*06∶04-DPB1*04∶01 haplotype had a single origin in Japan. 19,949 SNPs located on 6p were genotyped, and the average SNP density was 0.34 SNP/kb. The window and step sizes were 1 Mb and 200 kb, respectively. This analysis was performed using the SNP data from the five subject included in the SNP typing: three subjects were homozygous for the A*33∶03-C*14∶03-B*44∶03-DRB1*13∶02-DQB1*06∶04-DPB1*04∶01 haplotype and two subjects had the heterozygous genotypes of the A*33∶03-C*14∶03-B*44∶03-DRB1*13∶02-DQB1*06∶04-DPB1*04∶01 haplotype and the A*24∶02-C*07∶02-B*07∶02-DRB1*01∶01-DQB1*05∶01-DPB1*04∶02 haplotype and of the A*33∶03-C*14∶03-B*44∶03-DRB1*13∶02-DQB1*06∶04-DPB1*04∶01 haplotype and the A*24∶02-C*12∶02-B*52∶01-DRB1*15∶02-DQB1*06∶01-DPB1*09∶01 haplotype.

### Computer Simulation

To estimate the intensity of recent positive selection acting on DPB1*04∶01, a stochastic population genetic model (two-locus two-allele model) assuming both positive selection and random genetic drift was built and assessed. The diploid population size, *N*, was set to be 10,000 (i.e., 20,000 chromosomes). Four haplotypes carrying DPB1*04∶01 or non-DPB1*04∶01 alleles (designated by DPB1*X) at the *HLA-DPB1* locus and DQB1*06∶04 or non-*DQB1∶06:04* alleles (designated by DQB1*X) at the *HLA-DQB1* locus were used in this model. The frequencies of the DQB1*06∶04-DPB1*04∶01, DQB1*X-DPB1*04∶01, DQB1*06∶04-DPB1*X, and DQB1*X-DPB1*X haplotypes at generation *t* were denoted by *f*_1_(*t*), *f*_2_(*t*), *f*_3_(*t*), and *f*_4_(*t*), respectively. The current frequencies of the corresponding haplotypes in our study population were denoted by *f*_1_, *f*_2_, *f*_3_, and *f*_4_. A dominant selection was assumed for DPB1*04∶01 (i.e., relative finesses of DPB1*04∶01/DPB1*04∶01, DPB1*04∶01/DPB1*X, and DPB1*X/DPB1*X are 1, 1, and 1 - *s*, respectively). The initial haplotype frequencies were set as *f*_1_(*t*) = *z*, *f*_2_(*t*) = 0, *f*_3_(*t*) = (1−z)*f*_3_/(*f*_3_+ *f*_4_), and *f*_4_(*t*) = (1−z)*f*_4_/(*f*_3_+ *f*_4_). The recombination between *HLA-DPB1* and *HLA-DQB1* loci was assumed to occur at a rate of *c*. Since the recombination rate between *HLA-DQB1* and *HLA-DPB1* has been estimated to be between 0.004 and 0.012 [41], [42], a uniform recombination rate (*c*) within this range was used as a prior distribution. To estimate suitable parameter sets of *z*, *s*, and *c*, each value was drawn by a random number generator in every simulation run. The random numbers were between 0.0001 (i.e., 2/2*N*) and 0.005 (i.e., 100/2*N*) for *z*, between 0 and 0.1 for *s*, and between 0.004 and 0.012 for *c*.

Next, to evaluate the similarity between simulated and observed frequencies,
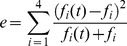
was calculated. As the simulated haplotype frequencies, *f*_1_(*t*), *f*_2_(*t*), *f*_3_(*t*), and *f*_4_(*t*), approaches values close to the observed frequencies, *f*_1_, *f*_2_, *f*_3_, and *f*_4_, the value of *e* approaches 0. The rejection method [18], [32], [33] was used to accept only simulation runs that resulted in (i) *e* of less than 0.01, (ii) *f*_1_(*t*) of not less than *f*_1_−0.01 nor more than *f*_1_+0.01, and (iii) *t* of not less than 92 nor more than 115 generations. A total of 2,500 runs were accepted. The mean and 95% credible interval of *s* were obtained from the 2,500 accepted runs.

## Supporting Information

Data S1 **Pairwise LD measures for individual HLA allele pairs.**
(XLSX)

Table S1 **Linkage Disequilibrium between pairs of HLA loci.**
(XLSX)

Table S2 **Estimated frequencies of 2-locus HLA haplotypes.**
(XLSX)

Table S3 **Estimated frequencies of 3-locus HLA haplotypes.**
(XLSX)

Table S4 **Estimated frequencies of 4-locus HLA haplotypes.**
(XLSX)

Table S5 **Estimated frequencies of 5-locus HLA haplotypes.**
(XLSX)
